# The Effects of Uniquely-Processed Titanium on Biological Systems: Implications for Human Health and Performance

**DOI:** 10.3390/jfb5010001

**Published:** 2014-01-03

**Authors:** David S. Rowlands, Sarah P. Shultz, Takahiro Ogawa, Wataru Aoi, Martin Korte

**Affiliations:** 1 School of Sport and Exercise, Massey University, 63 Wallace Street, Wellington 6140, New Zealand; E-Mail: s.p.shultz@massey.ac.nz; 2 University of California, Los Angeles, School of Dentistry, B3-088H CHS, 10833 Le Conte Ave, Los Angeles, CA 90095-1668, USA; E-Mail: togawa@dentistry.ucla.edu; 3 Graduate School of Life and Environmental Sciences, Kyoto Prefectural University, Shimogamo Hangi-cho 1-5, Sakyo-ku, Kyoto 606-8522, Japan; E-Mail: waoi@koto.kpu-m.ac.jp; 4 Zoological Institute, Technical University of Braunschweig, Spielmannstr. 7, Braunschweig D-38106, Germany; E-Mail: m.korte@tu-braunschweig.de

**Keywords:** Aqua Titan, action potential, long-term potentiation, tendon compliance, musculotendinous function, cell adhesion and growth, autonomic nervous system, pico-nanometer scale

## Abstract

Titanium is biocompatible and widely utilized in a variety of applications. Recently, titanium in pico-nanometer scale and soluble form (Aqua Titan) has expanded its use to applied human health and performance. The purpose of this article is to review the current evidence associated with specific physiological responses to Aqua Titan-treated materials. *In vitro* studies have shown that application of Aqua Titan can modify membrane potential and long-term potentiation in isolated hippocampal neurons, suggesting reduced pain memory as a possible mechanism for reported analgesia. Proximal contact with Aqua Titan-treated titanium increased gene expression, protein synthesis, cell growth and adhesion in normal cultured muscle and bone cells, suggesting application for Aqua Titan in clinical implant procedures and wound healing. Evidence for beneficial effects on neuromuscular control of muscle-tendon function and improvements in running economy in human athletes was seen when Aqua Titan-treated tape was applied to the human triceps surae following fatigue induced by prior strenuous exercise. Finally, behavioral responses and effects on the autonomic nervous system to environmental exposure suggest Aqua Titan may promote a mild relaxant, or stress-suppressive response. Together, data suggest exposure to Aqua Titan-treated materials modulates aspects of growth and function in neuronal and other musculoskeletal cells with possible benefits to musculotendinous recovery from exercise and to the systemic response to stress.

## 1. Introduction

Titanium metal’s low density, biocompatibility, and high strength-to-weight ratio make the element valuable in a variety of biological, orthopedic, dental, and tissue regenerative applications [1]. Recently, titanium has been dissolved in high-function water via a process involving the burning of a mixed gas of oxygen and hydrogen in high-pressure water and melting titanium in the combustible gas [2]. The unique titanium processing procedure generates picometer to micrometer sized particles of titanium termed Aqua Titan (trade-marked). When utilized as a dye, this form of titanium was found to readily bond to fabrics. As an integral component of the garment, tape or other materials, Aqua Titan has expanded possible biological and environmental application of titanium.

**Figure 1:**
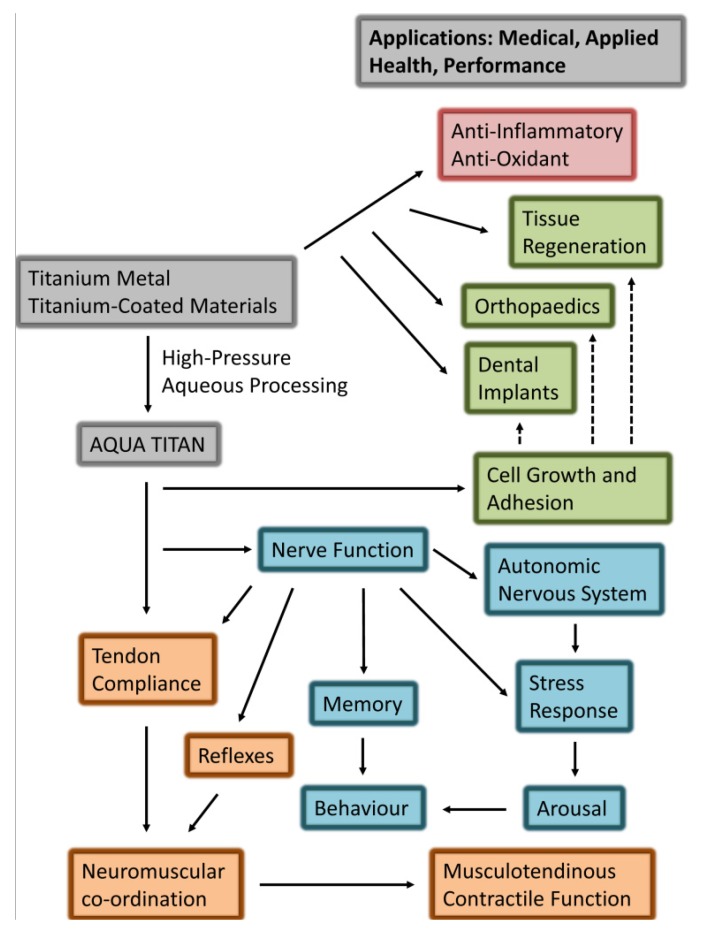
Summary of research-evidenced (solid arrows) and prospective (dashed arrows) effects of exposure to Aqua Titan-treated materials on physiological responses associated with health and performance phenotype. The responses highlight the biological effects of Aqua Titan-treated materials (grey) on cellular and tissue (green), neural and behavioral (blue), and musculoskeletal (orange) function.

Subsequent controlled investigations have generated evidence to suggest that exposure of cells and tissues to Aqua Titan-treated materials influence aspects of physiological function. The physiological responses to Aqua Titan may yield small-moderate standardized effect sizes on human health associated and performance-related phenotypes. Proximal location of materials coated with picometer to micrometer titanium particles reduced the resting membrane potential and action potential firing rate of pyramidal neurons [3] and increased alkaline phosphatase activity, mineral deposition, and bone-related gene expression in osteoblast cells [4]. These *in vitro* outcomes were consistent with other work showing titanium or titanium-coated materials to have anti-inflammatory and antioxidant effects, and promote nerve cell growth [5,6,7,8]. In humans, garments and tape permeated with Aqua Titan particles restored Achilles tendon stiffness and enhanced the short latency reflex response [9] with subsequent improvement in running economy [10] during recovery from strenuous hill running exercise in male athletes. Meanwhile, symptoms of stress were attenuated in office workers sleeping and working in rooms lined with Aqua Titan-treated materials [11]. The purpose of this article is to summarize the breadth of current research describing the biological effect of Aqua Titan-treated materials on cell and tissue function and animal and human behavior (Figure 1). 

## 2. Cellular Adhesion, Growth, and Function

Titanium and titanium alloys have been widely used in surgical implantation, primarily because of the bioavailability and tolerance of titanium within biological systems. Successful integration of titanium implants is often based on two properties: surface topography and surface chemical make-up [12,13,14]. The importance of these two properties to cellular response is varied between tissue types. 

**Figure 2:**
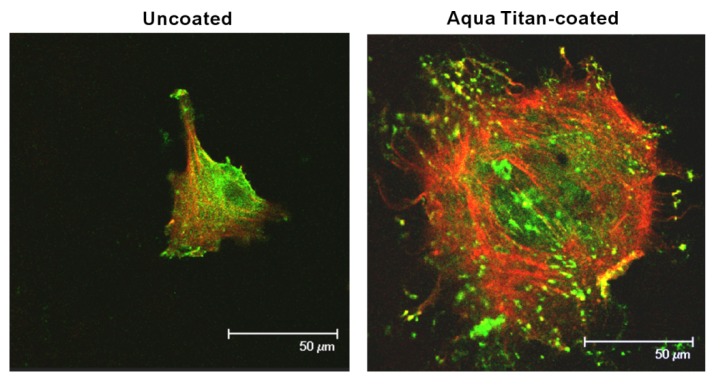
Initial spread, cytoskeletal arrangement, and establishment of focal adhesion of osteoblasts 24 h after seeding onto titanium surfaces with and without Aqua Titan-treated coating. Representative confocal microscopic images of cells stained with rhodamine phalloidin for cytoskeletal actin filaments (red) and anti-vinculin (green). The cells on Aqua Titan-treated titanium surfaces are clearly larger with their cytoplasmic projections more developed. The cells were also characterized with faster and more expression of actin and vinculin.

Altering the titanium surface characteristics played a significant role in the growth and behavior of normal skeletal muscle cells [13]. Titanium surface that was coated with Aqua Titan molten TiO_2_ nanoparticles promoted the aggregation and growth of the cells. The surface oxygen of the original titanium surface was increased by the Aqua Titan without changing surface topography, which seemed to be responsible for the increased bioactivity [13]. The modified surface chemistry of the coated titanium was also associated with greater upregulation in the expression of vinculin (Figure 2). As a focal adhesion protein, vinculin is important to the initiation of cell attachment and adhesion, as well as the establishment of cell shape and cytoskeleton development [15,16], improving structural and biomechanical properties of the cell [17,18]. Additionally, the gene expression of other important structural proteins (*i.e.*, collagen I and III, myosin, troponin T) were upregulated, which are not only structural, but promote differentiation and function of myoblasts [13]. In the study, the phenotypical modulation was examined only during the early stage of normal-cell culture, and further studies are required to establish the long-term effect of Aqua Titan as well as the effect at the tissue level.

The adhesion of osteoblasts that have been cultured on titanium is stronger than those cultured on a polymer-based material [19]. Similarly, multiple functions of bone-forming cells (osteoblasts) have been enhanced by Aqua Titan-treated titanium, relative to titanium alone [4]. Osteoblasts derived from bone marrow were cultured on titanium surfaces with and without Aqua Titan-treated coating. Aqua Titan-treated coating was pico-to-nano thin and thus improved osteoblast activity, regardless of the surface topography of the substrate [20]. The adhesion, gene expression, and mineralizing capability were substantially increased in normal cells on Aqua Titan-treated titanium surfaces. The increased function was correlated with the increased surface oxygen by Aqua Titan-treated coating. Titanium-based materials have been extensively used as various implant devices to immobilize and repair bone, connect bone with teeth, and regenerate bone. The increased osteoblastic function in the study implies the bone formation can be expedited and enhanced around Aqua Titan-treated biomaterials, providing a new possibility to improve the outcome of these clinical procedures. 

## 3. Neural Response

There is strong biocompatibility between titanium and nerves: TiO_2_ does not alter the structure or function of myelinated nerves [21], and direct contact between titanium metal implant and nerves did not lead to any neurotoxic effects [22]. Aqua Titan, when integrated into a tape applied to the skin, also proved to be non-toxic to neuronal tissue [3]. TiO_2_-rutile cultures showed good survival of CNS neurons, with typical development of axons [5]. Together, these data suggest a potential use for titanium in CNS implants. 

**Figure 3:**
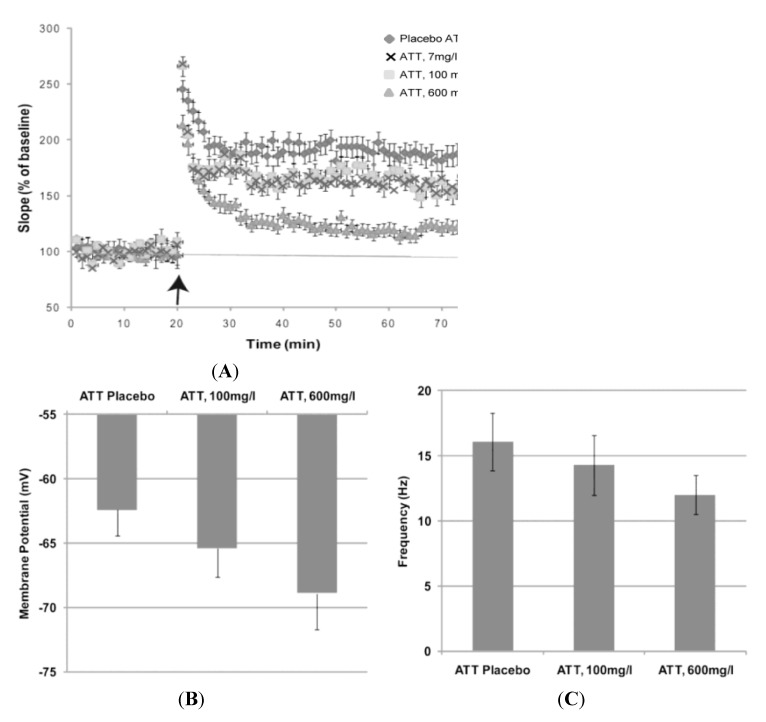
Effect of exposure to tape submerged and coated with three concentrations of Aqua Titan (ATT) and placebo on the following responses in mouse CA1 hippocampal pyramidal neurons: (**A**) long-term potentiation in response to 100 Hz stimuli; (**B**) resting membrane potential; and (**C**) the frequency of the action potential firing rate. Data represent the mean ± standard error. For all experiments, 12 hippocampal slices were used [3].

Anecdotal reports of a mild analgesia with skin surface application of Aqua Titan-treated tape have been investigated for potential neural response. A principle physiological pain mechanism in the central nervous system was examined *in vitro* for evidence of cellular response [3]. When compared to placebo tape, Aqua Titan impaired long-term potentiation (LTP) in mouse pyramidal neurons of the hippocampus, in a dose-sensitive manner (Figure 3). In this study, the tape was placed directly underneath neuronal tissue and still no side effects were observed. The experiments were repeated in pain processing pathways in the dorsal horn of the spinal cord. In the dorsal horn, Aqua Titan-treated tape also induced LTP, which is believed to be a component of the cellular mechanism for the implementation of central hyperalgesia [23]. The impairment was dependent on the concentration of Aqua Titan-treated fabrics (tapes), with the greatest impairment to LTP correlated to the highest concentration of Aqua Titan. Furthermore, the Aqua Titan-mediated effect on neurons was abolished with the positioning of a lead plate between the Aqua Titan-treated tape and the cell. These findings suggest that the uniquely prepared titanium has the ability to impair synaptic plasticity, which raises the potential for reduced sensory information about pain in the formation of a pain memory [3]. While previous research did not reveal evidence of any changes to compound action potentials when nerves came in contact with pure titanium implants [22], the chemical make-up of Aqua Titan dissolved in water allows for decreased resting-membrane potential and action-potential firing rates. Therefore, the important initial research suggests that Aqua Titan-treated tape may be useful in relieving pain without any neurotoxicity or side effects.

## 4. Musculotendinous and Skeletal Muscle Function

**Figure 4:**
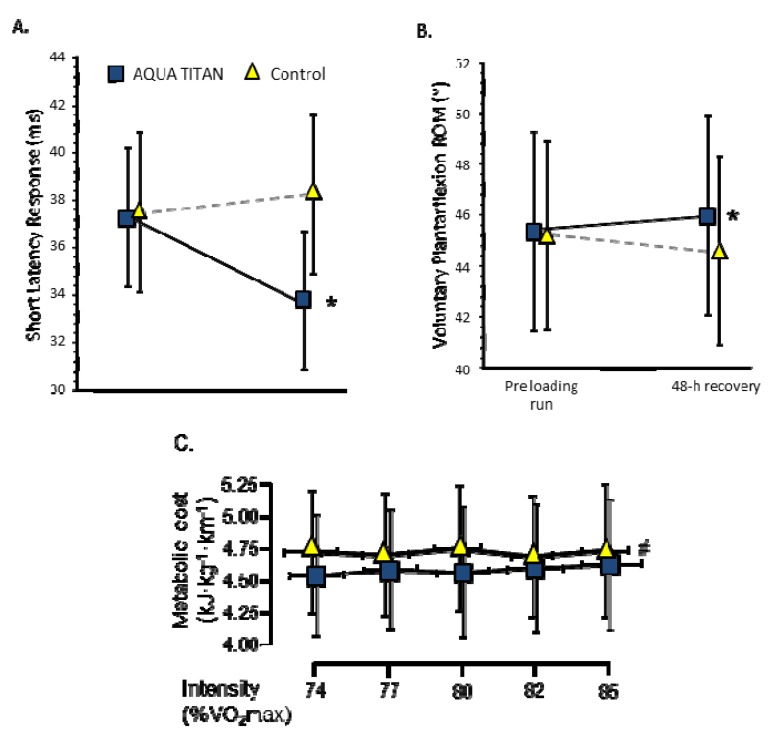
Effect of application of Aqua Titan-treated tape on the skin covering the triceps surae muscle-tendon complex and whole-body Aqua Titan-treated garments during 48 h recovery from strenuous running exercise on (**A**) the short latency response obtained from Achilles tendon tap; and (**B**) plantar flexor range of motion (ROM) from dynamometry [9]; and the on (**C**) the metabolic cost of subsequent running [10], respectively. Data represent mean ± standard deviation. Statistically significant differences from the control at *p* < 0.05, where * is the difference in the post-pre scores, and, # the difference for all run stages in the overall effect of treatment estimated via repeated measures linear mixed model ANOVA. Effect sizes were qualified as large for short latency response and small for range of motion (ROM) and metabolic cost.

Beyond surgical implants, Aqua Titan has been integrated into garments and material tapes and investigated for improved restoration of human musculoskeletal function following normal fatiguing exercise. Evidence drawn from recent research suggests that if the treated material is applied during recovery from strenuous running, there is faster restoration of joint range of motion [9,24], latency response time (reflex response), Achilles tendon stiffness [9], and running economy [10], relative to doubled-blind placebo controls (Figure 4). 

Anecdotal reports indicated reduced muscle and joint stiffness when Aqua Titan-treated tapes or patches were applied to the skin around the area of pain or tension, as a result of heavy exercise. Prior to investigation of Aqua Titan-treated garments on musculoskeletal function, possible mechanisms of physiological action were entirely speculative [24]. To shed light on possible physiological effect on muscle and tendon, whole-body polyester-nylon garments were treated with Aqua Titan. These garments were used to examine the effect of titanium application 48–72 h post-recovery on subsequent running economy [9,10,24]. Interestingly, increased active range of motion was observed not only in hip flexion and extension and ankle plantarflexion, but also in the shoulder [24], suggesting a global effect of Aqua Titan independent of response to musculotendinous stress. It was also proposed that the improved joint flexibility after exposure to Aqua Titan-treated material was a result of altered tendon function [24]; however, the neural, musculotendinous, or other physiological mechanisms for these changes were not identified. 

That skin-contact exposure to Aqua Titan-treated material could improve musculoskeletal function was substantiated by Rowlands [9]. Microtitanium impregnated adhesive tape was applied to the triceps surae during recovery from strenuous incline/decline treadmill running. Forty-eight hours into recovery, the fatigue-induced reduction in ankle plantarflexion range of motion and Achilles tendon stiffness was restored towards baseline, and there was an observed large effect size decrease in triceps surae short latency response time, suggesting faster neuromuscular reflex response. The short latency response is dependent on the balance of excitatory and inhibitory inputs from receptors, which subsequently modulates the excitability of motor neurons [25]. Decreased reflex response [9] suggests faster nerve conduction through a reflex arc might improve peripheral motor control. The peripheral response may, via afferent feedback networks, also influence central motor centers. Through such mechanisms, there is increased potential for alterations in postural balance or musculotendious unit contractile efficiency in cyclic movement [26]. 

Faster short latency response with Aqua Titan may improve gait efficiency independent of tissue adaptation. Reflex latency can modulate muscle-tendon complex stiffness by activating the spindle during stretch associated with contraction [27,28]. Tendon stiffness itself could also affect the contribution of afferent feedback [27]. Moreover, feedback from group II afferents in the muscle spindles and Golgi tendon organs were suggested to make an important contribution to running mechanical efficiency [29]. For tasks such as running, where the Achilles tendon does not undergo a substantial pre-stretch [30], it would be expected that maintenance of tendon stiffness would lead to less decrement in contractile-unit performance with fatigue. Furthermore, changes in tendon compliance or dynamic neuromuscular performance may be a key mechanism to account for improved contractile performance during running [26] and improved metabolic efficiency [10]. More investigation into regulatory influence of Aqua Titan on motor tasks and fine neural control of contractile stiffness is warranted.

The mechanism whereby dermal contact of Aqua Titan alters neuromusculotendinous function is unknown; however, several possible workings can be inferred. Runners reported increased thermal comfort with Aqua Titan-treated garments [24] suggesting altered thermal conductivity may be a mechanism. Titanium has a relatively high thermal conductivity of 5.8 W·m^−1^·K^−1^ for the most common titanium/aluminum/vanadium alloy (*vs*. 0.6 W·m^−1^·K^−1^ for water). The titanium particles in Aqua Titan-treated materials may, therefore, act as a heat sink for the tissue. Heat plays a major role in the function of physiological processes: in hyperthermia, muscles produce less force [31], tendon stiffness decreases [32], and nerve performance is impaired [33]. Therefore, it is necessary to examine the effect of Aqua Titan on skeletal muscle and tendon temperature. Specifically, it is important to understand if by maintaining a more consistent temperature during contraction, altered material thermal conductivity could explain the improvements in motor reflex latency, ROM, and maintenance of tendon stiffness [9,24]. 

A second possible mechanism to explain the Aqua Titan effect on recovering musculoskeletal function and improved restoration of running metabolic efficiency is faster repair or regeneration of damaged skeletal muscle tissue. Damage to skeletal muscle myofibers follows strenuous eccentric exercise [34,35]. Such tissue insult could impair stride efficiency by reducing stride length [34], range of motion [36], and knee extensor torque [35] with consequent impaired contractile function and reduced running economy. Damage to Type-I muscle fibers, which are heavily recruited during endurance running, forces increased relative recruitment of Type-II fibers. Type-II specific myosin adenosine triphosphatase isoforms require 1.6 to 2.1 times more adenosine triphosphates per unit force production than Type-I [37] and therefore requires proportionately higher oxidative phosphorylation. Lower minute ventilation (4%) was observed during sub-maximal exercise with Aqua Titan, offering some physiological support for relatively higher Type-II fiber recruitment profile [38] because minute ventilation is coupled to muscle contraction by locomotor muscle afferents [39]. Histologically, damaged muscle undergoes repair and adaptive regeneration mediated by inflammatory process associated with wound healing responses followed by enhanced myogenesis [40]. As mentioned earlier, Ishizaki *et al*. [13] reported that in cell culture, Aqua Titan-treated rubber upregulated the expression of myofibril components and accelerated myocyte and osteoblast growth. If such a mechanism was also active *in vivo*, accelerated skeletal muscle cellular and tissue regeneration could be another contributing mechanism for faster restoration of musculoskeletal contractile function following damaging exercise.

## 5. Behavioral and Psychological Responses

**Figure 5:**
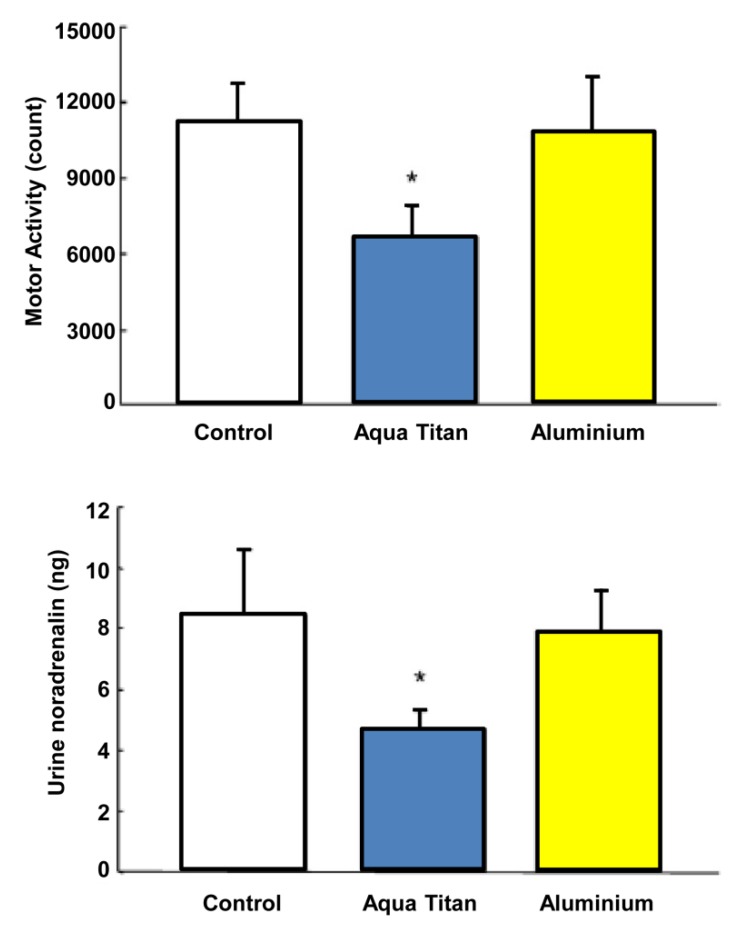
Effect of Aqua Titan treated cages on spontaneous movement following cage change and urinary noradrenalin content. Spontaneously activity was assessed in the light period as the integrated number of single animal motor counts per 12 h period. Noradrenalin content was the total content collected over a 24 h measurement. Data represent mean ± standard error *n* = 8. * Statistically significant differences from the control at *p* < 0.05 [41].

Local exposure to wall and flooring materials and garments treated with Aqua Titan has been shown to have some impact on some physiological and behavioral responses to local environmental stress. Aoi *et al*. [41] evaluated autonomic nervous system activity in mice housed in cages with Aqua Titan-treated rubber sheets attached to the inside and outside walls, relative to rubber sheets free of titanium (placebo). The Aqua Titan treatment led to a predominance of parasympathetic over sympathetic nerve activity, as assessed with power spectral analysis of heart rate variability and by a reduction in urinary noradrenaline concentration (Figure 5). The titanium-caged mice also presented decreased spontaneous activity in response to housing changes during their sleeping (*i.e.*, prolonged the sleeping period); the decrease was not found when the Aqua Titan-treated rubber sheets were wrapped in aluminum. Housing change normally excites mice leading to increased movement around cages and less sleep [42]. These observations suggest that exposure to Aqua Titan in the proximal environment has a relaxant effect via lowering aspects of the physiological stress response and attenuating stress-associated rodent behavior.

The acquisition of early evidence for a similar relaxant effect in humans was provided recently by the same research group [11]. A randomized cohort of 24 male office workers in desk jobs with daily emotional stress slept in a room with the inside of the walls and floor coated with Aqua Titan-treated rubber sheets in the same manner as in the rodent study [41], and compared against titanium-free placebo run in double-blind conditions. Workers reported to the clinic and blood samples were collected for baseline measurements. After a full work-day subjects returned to the clinic and were housed in individual experimental rooms overnight. The work-sleep pattern was repeated over a 5 day period. Morning serum adrenocorticotrophic and cortisol concentrations increased significantly from baseline on day 4 by 81% and 36%, respectively, but altered less in the Aqua Titan group. Autonomic nervous system activity was evaluated by heart rate variability. The power spectral analysis of time between ventricular depolarizations (R–R interval data) showed a significant elevation in the high-frequency power spectral ratio in subjects housed in Aqua Titan rooms during the awake period, relative to baseline (14%–27%). Conversely, no significant changes over time were observed in the placebo group in either the awake or sleeping period. These data suggest Aqua Titan-treated rooms induced dominance of the parasympathetic over sympathetic nervous system activity, which also strongly supports the corticoid results. On closer examination, the spectral analysis indicated gradual elevation of sympathetic nervous system activity during daytime office work over the course of the study in the placebo group but not in the Aqua Titan group, which maintained basal level. Using the Profile of Mood States (POMS), it was observed that 5 days within the Aqua Titan-treated room led to a moderate effect size reduction in the anger-hostility score, relative to placebo, and an increase in the vigor-activity score in the treatment group, while factors tension–anxiety and anger–hostility were decreased [11]. These observations suggest that the Aqua Titan surroundings during night time leads to moderate suppression of psychological stress associated with autonomic nerve regulation, not excess relaxation in the daytime. As there was distance between the sleeping spaces of the subjects and the Aqua Titan-treated walls and floor in this study, it appears that Aqua Titan may exert effects even when not in direct contact with the body. This is in agreement with the *in vitro* study by Korte [3], which found that Aqua Titan-treated tape could affect pyramidal neurons when placed under the culture dishes. 

Some psychological effect of Aqua Titan exposure was observed in response to wearing Aqua Titan-treated garments during 5 days’ recovery from simulated high-intensity soccer play in trained men [24]. On day 3 of recovery, subjects reported lower negative Daily Analyses of Life Demands in Athletes (DALDA) symptoms of stress, but elevated anger and esteem in the POMS assessment. Moreover, participants reported elevated feeling of overall well-being under Aqua Titan treatment on days 3 and 4, but outcomes were unclear on days 2 and 5. The magnitude of the stress-suppressive effects of Aqua Titan [11,24] could be considered relatively modest, but is within the range (4% to 66%) reported for studies using the POMS test in subjects who used exercise, meditation, and dietary supplement to improve mood [43].

Together, there is some evidence to support anecdotes for a behavioral impact of proximal exposure to Aqua Titan-treated materials. The autonomic nervous system responses are relevant to health because they are integral to cardiovascular, neuroendocrine, pain, and behavioral responses essential for survival, and are therefore implicated in diseases, such as, hypertension and obesity [44]. In addition, sleeplessness is related to autonomic nerve system disturbance and high psychological stress causing serious problems in daily living and the work place [45,46]. 

## 6. Conclusions

The effects of Aqua Titan on neuronal and sympathetic nerve function, relaxation in mice and humans, and restorative impacts on musculotendinous function support several possibilities for improved health and performance. The lowering of long-term potentiation and change to resting membrane potential in pyramidal neurons with Aqua Titan-treated tape suggests not only a mechanism for reduced pain memory, but also possible altered nerve function in general. Aqua Titan may also regulate joint stiffness and range of motion via altered neuromuscular pathways. It is possible, therefore, that a similar effect of Aqua Titan on the membrane potential of sympathetic and parasympathetic nerves could change nerve activity, leading to the alleviation of emotional stress, and raise the prospect of effects on other autonomic nervous system regulated physiological responses (e.g., blood pressure and flow). Evidence for improved cellular growth and musculoskeletal efficiency, suggests Aqua Titan may enhance muscle recovery from prior strenuous fatiguing exercise. Further research is required to determine the magnitude and clinical effects of longer-term exposure to living and working spaces and to wearable garments and tapes treated with Aqua Titan. Research should also determine the physical and physiological mechanisms, which will expand the evidence base for application.
